# Exploring the nutritional status in adults with chronic schizophrenia: a cross-sectional study in Ecuador

**DOI:** 10.3389/fnut.2025.1658703

**Published:** 2025-09-05

**Authors:** José Alejandro Valdevila Figueira, Alfonso Daniel Silva Ochoa, Luz María Valencia Erazo, María Gracia Madero Dutazaka, Galya Bigman, Indira Dayana Carvajal Parra

**Affiliations:** ^1^Instituto de Neurociencias de Guayaquil, Guayaquil, Ecuador; ^2^Universidad Ecotec, Samborondón, Ecuador; ^3^Red de Investigacion en Psicologia y Psiquiatria (RIPYP), Guayaquil, Ecuador; ^4^Polytechnic University, ESPOL, Campus Gustavo Galindo, Guayaquil, Ecuador; ^5^University of Maryland, Baltimore, MD, United States

**Keywords:** nutrition status, prevalence, schizophrenia, mental conditions, vitamin D

## Abstract

**Introduction:**

Schizophrenia (SCZ) and other related factors could be associated with specific nutritional problems. Some serum biomarkers could be involved in the clinical presentation of psychotic disorders. These individuals could have significantly lower bone mineral density (BMD) and a higher prevalence of osteoporosis comparatively.

**Objective:**

The purpose of our study was to assess the association of key elements of the nutritional status between patients with SCZ and other mental illnesses to promote effective treatment plans.

**Methods:**

This was an observational, cross-sectional study with convenience sampling. The sample was divided into two groups: SCZ (S) (*n* = 66) and no SCZ (NS) (*n* = 47). We included 113 adults aged from 22 to 85 years admitted to the Institute of Neurosciences of Guayaquil (INC) residency. Anthropometric and body composition indicators were analyzed. Blood samples were collected using appropriate venipuncture techniques, ensuring aseptic conditions and minimizing hemolysis. Wilcoxon rank sum test, two-sample t test, Fisher’s exact test, and linear regression were applied to assess variables among groups.

**Results:**

The median BMI was 24.14 kg/m^2^. Visceral fat and serum creatinine were significantly higher in the S group. The prevalence of anemia, low vitamin D, low HDL, high total cholesterol, and low creatinine was 64.60, 68.14, 22.12, 10.62, and 30.97%, respectively. BMI, age, and body fat jointly influenced creatinine (*p* = 0.03265), while BMI and age were strongly associated with visceral fat (*p* < 0.001). No significant associations were found between CRP and body fat or BMI.

**Conclusion:**

The nutritional treatment in these patients should aim to prevent and treat anemia, low vitamin D, low HDL, high total cholesterol, low bone mass, and low creatinine serum levels in these groups of patients. Visceral fat and body fat percentage tend to increase with aging and should be monitored carefully. The treatment should be multidisciplinary. More studies are needed to better understand this interplay.

## 1 Introduction

Schizophrenia (SCZ) and antipsychotic use are associated with clinically significant weight gain and subsequent increased mortality (1). The prevalence of obesity and other modifiable cardiometabolic risk factors (e.g., smoking, type-2 diabetes mellitus (T2DM), hypertension, dyslipidemia, and metabolic syndrome) is higher in individuals with SCZ, with relative risks for each factor ranging between 1.5 and 5 compared with the general population (2).

The weighted lifetime prevalence of SCZ varies by country and region between 0.3 and 1.3% (3). Chronic forms of SCZ experience marked cognitive impairment with multiple negative symptoms that invalidate social behavior (4).

SCZ most frequently appears in late adolescence or between 20 and 30 years of age, and in men it usually manifests earlier than in women, despite not being as common as many other mental disorders (5). People with SCZ are two to three times more likely to die prematurely than the general population, often from cardiovascular, metabolic, or infectious diseases (6).

1,25 (OH)_2_D_3_ (vitamin D) is well-recognized as a neurosteroid that modulates multiple brain functions. Vitamin D could be involved in the clinical presentation of psychotic disorders, and the correlation has been implicated in meta-analysis (7–9). A growing body of evidence indicates that vitamin D plays a pivotal role in brain development, neurotransmission, neuroprotection, and immunomodulation (7). Accumulating data show that there may be an association between vitamin D deficiency and SCZ (9), and also, low maternal vitamin D was proposed as a risk factor for SCZ almost two decades ago (10). However, the precise molecular mechanisms by which vitamin D exerts these functions in the brain are still unclear (7).

Multiple risk factors, such as genetic, environmental factors (9), as well as psychiatric patients’ lifestyle (11) and antipsychotic drugs (12) may contribute to vitamin D deficiency, leading to a high risk of general dysfunction (13).

Patients with SCZ and bipolar disorder (BD) had significantly lower bone mineral density (BMD) and significantly higher prevalence of low bone mass and osteoporosis compared with healthy controls. Binary regression analysis showed that SCZ or BD was an independent risk factor for low bone mass and osteoporosis. In addition, sex, age, and bone turnover markers also influenced the occurrence of low bone mass and osteoporosis (14). A multiple regression analysis by Carswell et al. found an increased risk of low BMD associated with lower folate and glycated hemoglobin levels, older age, and higher serum ferritin and follicle-stimulating hormone (FSH) levels. In female patients, BMD was mainly associated with age and serum hormones (FSH and testosterone), whereas BMD in male patients was mainly related to age, trace elements (serum ferritin and 25-hydroxyvitamin D [25(OH)D]), and parathyroid hormone (15).

There is an increased risk of chronic kidney disease (CKD) amongst people who have severe mental illness compared with those who do not. However, people who have severe mental illness and CKD are less likely to receive specialist nephrology care (16). Individuals with severe mental illness, including conditions such as SCZ and BD, are at a higher risk of developing CKD. Higher incidences of CKD in this population can be partially explained by known risk factors, such as treatment with lithium carbonate and higher rates of cardiovascular disease (17).

Although nutritional status plays a critical role in health outcomes, there is a paucity of data on the nutritional profiles of Latin American patients diagnosed with SCZ. Most existing research has focused on North American and European populations, leaving a gap in understanding dietary intake, anthropometric parameters, and micronutrient deficiencies in Latin American contexts. This deficiency in evidence hinders the development of personalized nutritional interventions and public health strategies. Comprehensive studies are urgently needed to elucidate nutritional status and inform robust, culturally appropriate, and evidence-based guidelines for this vulnerable demographic.

Our hypothesis is that individuals with SCZ will exhibit increased visceral adiposity and reduced serum levels of 25-hydroxyvitamin D [25(OH)D] compared with those without SCZ. The purpose of our study was to analyze the nutritional status of patients with SCZ and other mental illnesses.

## 2 Method

### 2.1 Study design

This was an observational, cross-sectional with convenience sampling study. The sample was divided into two groups: SCZ (S) (*n* = 66) and no SCZ (NS) (*n* = 47).

### 2.2 Participants

We included 113 adults aged from 22 to 85 years admitted to the Institute of Neurosciences of Guayaquil (INC) residency. The INC has dietitians and a food service under contract. Participants aged more than 60 years were labeled as older adults. They were diagnosed with either chronic SCZ (S group) or other mental conditions (NS group), such as intellectual disability, dementia, brain injury, epilepsy, and meningitis. All patients were taking psychotropic medication at the time of the study, highlighting the use of carbamazepine, pregabalin, quetiapine, chlorpromazine, biperiden, levomepromazine, and some other medications depending on comorbidity with organic pathologies such as diabetes mellitus, arterial hypertension, and hypothyroidism (levothyroxine, metformin, carvedilol, and losartan). Other medications, such as clozapine, are not discussed in this section because they are not included in the National Essential Medicines List and, consequently, are not administered to this patient population. Patients are placed in separate areas for men and women for clinical management purposes, in accordance with the institution’s structural design.

Adult subjects, with a diagnosis of long-standing mental illnesses (5 years or more of continuous evolution), of both sexes, admitted to the INC residence area, were included in the study. Subjects with little predisposition to collaborate in the study, with complications inherent to the evolution of the mental illness and/or physical limitations due to other pathologies, and those who left the residence for any reason during the data collection process were excluded from the study. This study was approved by the Human Research Ethics Committee of the Luis Vernaza Hospital of the Guayaquil Charity Board (code 09-EO-CEISH-HLV-2023) and conducted in full compliance with the Declaration of Helsinki. Participant recruitment began on 7 August, 2023, 1 month after ethical approval was granted in July 2023, and continued for 4 months, concluding on 7 December 2023.

### 2.3 Nutritional assessment

Height, weight, hand grip, and body composition were measured. Body composition included fat mass, muscle mass, visceral fat, and water percentage. Height was measured with the SECA 213 portable stadiometer. Weight and body composition were analyzed using the TANITA BC-420 MA body composition analyzer scale. Grip strength was measured three times with the patient seated, with the arm flexed at 90°, and on both arms. The highest value was recorded. It was measured with the FBA_EH101 CAMRY dynamometer.

The Malnutrition Screening Tool (MST) and the Global Leadership Initiative on Malnutrition (GLIM) criteria were used to identify nutritional risk and malnutrition, respectively. The MST is a quick and easy tool that helps identify people at risk of malnutrition and consists of two questions. GLIM criteria are a two-step process for diagnosing malnutrition. The criteria include phenotypic and etiologic criteria and are used to assess a patient’s nutritional status.

### 2.4 Blood biochemistry

For the collection of blood samples, the identity of the patient and the correspondence of their data with the indication of the tests to be performed, and the identification labels on the tubes were checked. EDTA tubes preserved cellular integrity for accurate, complete blood counts, while serum separator tubes facilitated clot formation and clean separation for dependable analysis of hepatic enzymes and lipid profiles. The collection was performed in a safe and respectful environment through venipuncture, respecting the privacy of the patient and the confidentiality of their data. The patient was informed of the procedure to be performed, and his or her cooperation was requested. With verbal consent, a fasting blood sample was taken, following the national regulations for this type of examination according to the Ministry of Health. Patients were placed in a sitting position with the arm chosen for the puncture in hyperextension, and aseptic and antiseptic conditions were ensured to minimize hemolysis according to current national regulations.

Validated assays were used for the following analytes: creatinine, aspartate transaminase (AST or GOT), alanine transaminase (ALT), gamma-glutamyl transferase (GGT), hemoglobin, HDL, vitamin D, white blood cells, and C-reactive protein (CRP). We considered the Endocrine Society clinical guideline for the vitamin D levels.

Venous blood samples (10 mL) were collected for laboratory analyses. Hemoglobin was determined using the photometric Sodium Lauryl Sulfate method (SLS-HGB, cyanide-free; Roche Diagnostics) on a Sysmex 1000 analyzer. Serum AST, ALT, and GGT activities were measured by photometry using Roche reagent kits 800, 800, and 500, respectively, on a COBAS 8000 analyzer. Creatinine was analyzed photometrically with the Roche reagent kit 2500 (COBAS 8000). HDL cholesterol was measured photometrically using the Roche reagent kit 800 on a COBAS 8000. Vitamin D was quantified by electrochemiluminescence immunoassay (ECLIA) with Roche reagent kit 100 on a COBAS 8000. White blood cell count was obtained by fluorescent flow cytometry (Sysmex 1000, Roche). CRP was determined by turbidimetry using the Roche reagent kit 500 on a COBAS 8000 analyzer. All assays were performed according to the manufacturer’s validated protocols.

The eGFR was not considered for inclusion because certain required variables, such as age, were not reliably available. Additionally, factors common to our sample—including altered muscle mass, psychotropic medication effects, and variability in dietary protein intake—may confound the relationship between serum creatinine and true glomerular filtration. These potential sources of bias could lead to inaccurate eGFR estimates. Therefore, we relied exclusively on direct serum creatinine measurement as a robust marker of renal function, deferring incorporation of estimated GFR.

### 2.5 Statistical analysis

The Kolmogorov–Smirnov test was applied to assess the normality of the dataset. Since age, BMI (body mass index), body fat, creatinine, HDL (high-density lipoprotein), hemoglobin, AST, ALT (alanine transaminase), GGT (γ glutamyl transferase), CRP (C-reactive protein), bone mass, and white blood cell counts were not normally distributed, the Wilcoxon rank-sum test was used to compare these variables between groups.

The two-sample t-test was used to determine significance for height, weight, hand grip, total water, vitamin D, and muscle mass between groups. Fisher’s exact test was used to analyze ethnicity, BMI, body fat interpretation, and SCZ diagnosis. Linear regression was applied to analyze the relationship between age, creatinine, visceral fat, CRP, and BMI. The statistical significance was set as *p* < 0.05. The statistical analyses were conducted using the R statistical computing platform, R version 4.3.1.

## 3 Results

### 3.1 Baseline characteristics

The baseline characteristics of the sample are presented in Table 1. Statistically significant differences were found in age (S: 56.76 ± 11.40 years; NS: 50.26 ± 13.56 years; *p* = 0.005) and hand grip strength (S: 19.11 ± 10.15 kg; NS: 12.64 ± 8.70 kg; *p* < 0.001) between groups. The lower hand grip in the NS group could be related to neuromotor impairment (e.g., brain injury), so this finding should be interpreted with caution. No significant differences were observed for other baseline characteristics. The sample included participants from various ethnic groups; although most participants self-identified as white according to INEC categories, there were no significant group differences in ethnicity (Table 1).

**Table 1:** Baseline characteristics of our participants, all and by the S and NS groups.

Characteristic	All participants (*n* = 113)	S (*n* = 66)	NS (*n* = 47)	*p*-value
Age	54.053 ± 12.700	56.758 ± 11.395	50.255 ± 13.564	0.005
Sex				
Male	77 (68.14)	47 (71.2)	30 (63.8)	0.421
Female	36 (31.86)	19 (28.8)	17 (36.2)	
Ethnicity				
Afro-Ecuadorian	4 (3.54)	3 (4.5)	1 (2.1)	0.8741
Indigenous	4 (3.54)	2 (3)	2 (4.3)	
Mixed-race	7 (6.19)	4 (6.1)	3 (6.4)	
Montubio	3 (2.65)	1 (1.5)	2 (4.3)	
White	95 (84.07)	56 (84.8)	39 (83)	
Anthropometry				
Height (cm)	159.4 ± 9.39	160.21 ± 8.17	158.17 ± 10.87	0.2799
Weight (kg)	63.72 ± 12.76	64.48 ± 10.77	62.68 ± 15.19	0.4879
Low weight (<18.5 kg/m^2^), %	3 (2.65)	1 (1.5)	2 (4.3)	0.8476
Normal weight (18.5–24.9 kg/m^2^)	61 (53.98)	35 (53)	26 (55.3)	
Overweight (25–29.9)	30 (26.55)	18 (27.3)	12 (25.5)	
Obesity (≥30 kg/m^2^)	19 (16.81)	12 (18.2)	7 (14.9)	
Nutritional status				
Hand grip, kg	16.41 ± 10.06	19.11 ± 10.15	12.64 ± 8.7	<0.001
MST nutritional risk				
Not at risk	113 (100)	66 (100)	47 (100)	N/A
At risk	0 (0)	0 (0)	0 (0)	
Malnutrition				
No malnutrition	113 (100)	66 (100)	47 (100)	N/A
Moderate malnutrition	0 (0)	0 (0)	0 (0)	
Severe malnutrition	0 (0)	0 (0)	0 (0)	

Values are in number of cases (%), mean ± SD. *p* means *p*-value. This number indicates significant differences between schizophrenia (S) and no schizophrenia (NS) groups.

### 3.2 Serum biochemical profile

The stepwise multiple linear regression for serum creatinine (Table 2) showed that BMI (*p* = 0.811), age (*p* = 0.754), and body fat percentage (*p* = 0.072) were not individually significant predictors. However, the overall model was statistically significant (*p* = 0.033), indicating a combined effect of these predictors on creatinine levels.

**Table 2:** Stepwise multiple linear regression analysis on creatinine.

Independent variable	*B*	SE	*β*	*p*-value	95% CI Lower	95% CI Upper	VIF
Constant	0.845	0.140		<0.001	0.568	1.123	
BMI	0.001	0.006	0.041	0.811	−0.011	0.014	3.460
Age	0.000	0.001	0.030	0.754	−0.002	0.003	1.059
Body fat %	−0.006	0.003	−0.312	0.072	−0.013	0.001	3.460

SE, standard error; *β*, standardized partial regression coefficient; CI, confidence interval; VIF, variance inflation factor.

In the total sample, the prevalence of anemia, low vitamin D (<30 ng/mL), low HDL, high total cholesterol, and low creatinine was 64.6, 68.1, 22.1, 10.6, and 31.0%, respectively (Table 3). Between-group comparisons showed no significant differences for most serum variables, except for low creatinine, which was more frequent in the NS group (*p* = 0.017; Table 3).

**Table 3:** Prevalence of serum abnormalities in all participants, S and NS groups.

Variable	All participants (*n* = 113)	S (*n* = 66)	NS (*n* = 47)	*p*-value
Prediabetes	7 (6.2)	5 (7.6)	2 (4.3)	0.104
Anemia	73 (64.6)	41 (62.1)	32 (68.1)	0.360
Vitamin D, ng/mL				
Deficient (≤20 ng/mL)	43 (38.1)	24 (36.4)	19 (40.4)	0.682
Insufficient (21–29 ng/mL)	34 (30.1)	22 (33.3)	12 (25.5)	
Optimal (≥30 ng/mL)	36 (31.9)	20 (30.3)	16 (34)	
Low HDL	25 (22.1)	15 (22.7)	10 (21.3)	1.000
High total cholesterol	12 (10.6)	7 (10.6)	5 (10.6)	0.615
Low creatinine	35 (31.0)	15 (22.7)	20 (42.6)	0.017
Low uric acid	53 (46.9)	28 (42.4)	25 (53.2)	0.477

Values are in number of cases (%), mean ± SD. *p* means *p*-value. This number indicates significant differences between schizophrenia (S) and no schizophrenia (NS) groups.

### 3.3 Body composition and nutritional biomarkers

Significant differences in body composition were observed only for visceral fat, with higher values in the S group (S: 9.39 ± 3.53; NS: 7.45 ± 4.00; *p* = 0.003; Table 4). No significant differences were found in other body composition parameters or most biochemical markers, except for serum creatinine, which was higher in the S group (S: 0.74 [0.68–0.92] mg/dL; NS: 0.67 [0.62–0.78] mg/dL; *p* = 0.014; Table 4).

**Table 4:** Body composition and blood biomarkers related to nutritional status by the S and NS groups.

Variable	All participants (*n* = 113)	S (*n* = 66)	NS (*n* = 47)	*p*-value
Body composition				
Body fat	21.4 [14.08–29.3]	23.4 [14.88–29.8]	19.4 [15.10–28.5]	0.371
Low body fat	12 (10.6)	5 (4.4)	7 (6.2)	0.419
Healthy body fat	56 (49.6)	31 (27.4)	25 (22.1)	
Overweight body fat	34 (30.1)	23 (20.4)	11 (9.7)	
Obesity body fat	11 (9.7)	7 (6.2)	4 (3.5)	
Visceral fat	8.58 ± 3.84	9.39 ± 3.53	7.45 ± 4.00	<0.01
Total water, %	54.39 ± 7.75	53.80 ± 7.29	55.21 ± 8.36	0.353
Muscle mass, %	45.52 ± 6.79	45.98 ± 6.38	44.87 ± 7.33	0.405
Bone mass, kg	2.4 [2.2–2.78]	2.4 [2.23–2.6]	2.4 [2.15–2.7]	0.409
Low bone mass	85.8%	90.9%		
Blood biomarkers				
Creatinine, mg/dL	0.72 [0.64–0.89]	0.74 [0.68–0.92]	0.67 [0.62–0.78]	0.014
AST (GOT), U/L	23 [20–29]	23 [19–29.75]	23 [20–28]	0.637
ALT, U/L	21 [16–26]	21 [16–24]	20 [15–27.5]	0.868
GGT, U/L	31.34 [16.3–53.5]	30.5 [16.22–50]	36.9 [17.10–53.7]	0.798
Hemoglobin, g/dL	12.7 [11.8–13.5]	12.5 [11.8–13.60]	12.8 [11.65–13.35]	0.991
HDL, g/dL	55 [45–64]	54 [44.25–64]	57 [47.5–64.5]	0.454
Vitamin D, ng/mL	24.72 ± 9.19	24.65 ± 8.29	24.82 ± 10.42	0.929
White blood cells, ×10^9^/L	6.15 [4.83–7.41]	6.12 [4.94–7.45]	6.16 [4.75–7.12]	0.930
CRP	2.38 [0.98–6.20]	2.51 [1.02–6.28]	2.35 [0.95–5.78]	0.477

Data are presented as number (%), mean ± SD, or median [IQR]. Statistical significance was set at *p* < 0.05. AST, aspartate transaminase; ALT, alanine transaminase; GGT, gamma-glutamyl transferase; HDL, high-density lipoprotein; CRP, C-reactive protein. Body fat ranges vary by sex and age. Fisher’s exact test was used to analyze body fat interpretation and SCZ diagnosis. Schizophrenia (S) and no schizophrenia (NS) groups.

In the regression analysis for visceral fat (Table 5), BMI (*p* < 0.001), and age (*p* < 0.001) were significant predictors, while body fat percentage was not (*p* = 0.944). The overall model showed a strong association of BMI and age with visceral fat levels (*p* < 0.001).

**Table 5:** Stepwise multiple linear regression analysis on visceral fat.

Independent variable	*B*	SE	*β*	*p*-value	95% CI Lower	95% CI upper	VIF
Constant	−12.142	1.574		<0.001	−15.263	−9.023	
BMI	0.400	0.070	0.568	<0.001	0.262	0.539	3.460
Age	0.198	0.017	0.653	<0.001	0.165	0.230	1.059
Body fat %	−0.003	0.038	−0.007	0.944	−0.078	0.073	3.460

SE, standard error; *β*, standardized partial regression coefficient; CI, confidence interval; VIF, variance inflation factor.

### 3.4 Inflammatory markers and figure-based analyses

**Figure 1:**
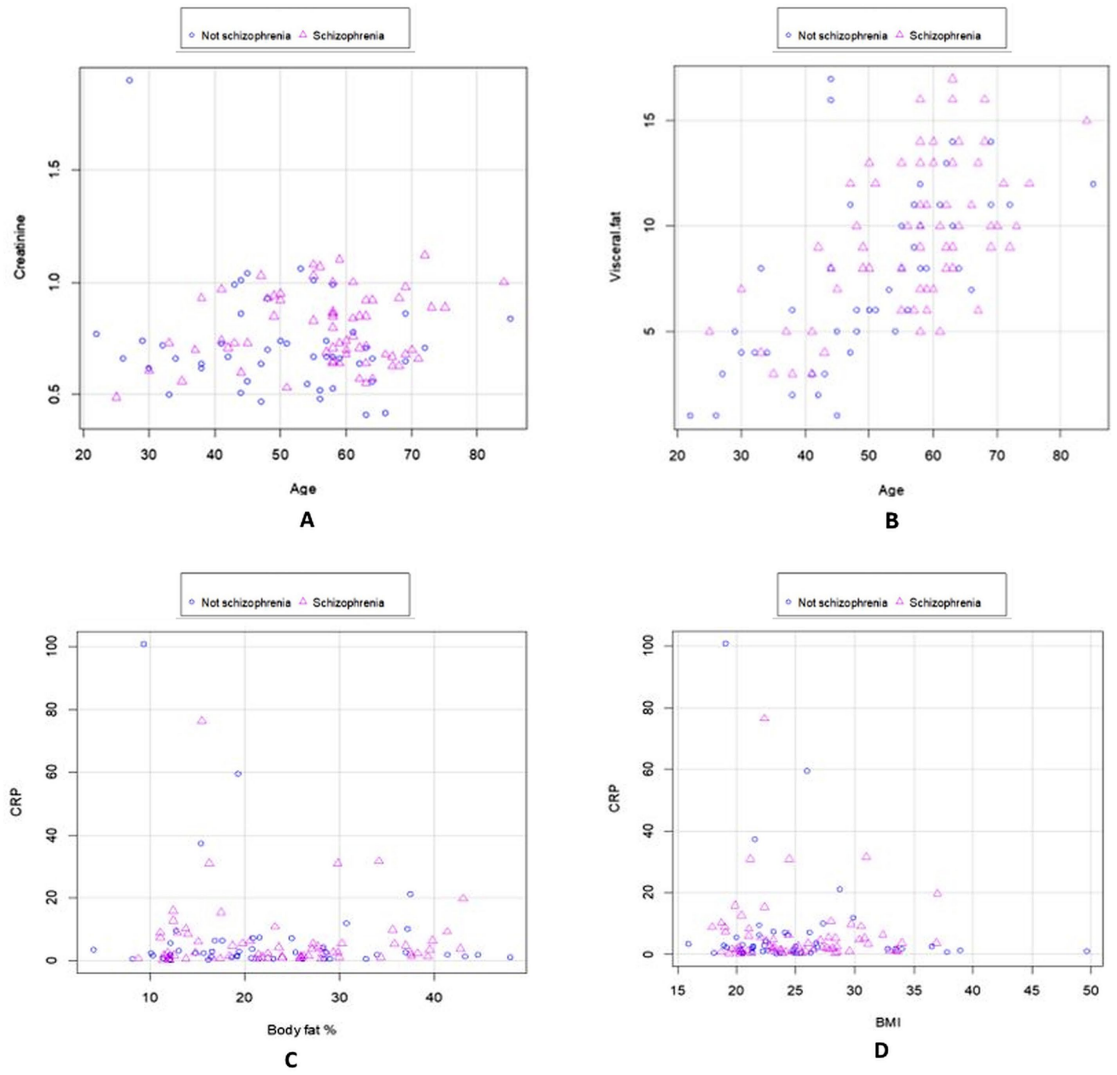
Scatter plots show the comparison of biochemical and anthropometric variables between schizophrenia and non-schizophrenia. Clinical diagnosis, creatinine, and age **(A)**; Clinical diagnosis, visceral fat, and age **(B)**; CRP, body fat, and diagnosis **(C)**; and CRP, BMI, and diagnosis **(D)**.

For C-reactive protein (CRP), none of the individual predictors (BMI, age, or body fat percentage) was significant, and the overall regression model was not significant (*p* = 0.687; Table 6). According to Figure 1A, the relationship between creatinine and age was not significant (*p* = 0.952), and no marked decrease in creatinine was observed after 60 years of age.

**Table 6:** Stepwise multiple linear regression analysis on CRP.

Independent variable	*B*	SE	*β*	*p*-value	95% CI Lower	95% CI Upper	VIF
Constant	8.213	10.244		0.424	−12.090	28.516	
BMI	−0.017	0.455	−0.006	0.971	−0.919	0.885	3.460
Age	0.044	0.108	0.040	0.682	−0.170	0.259	1.059
Body fat %	−0.148	0.248	−0.106	0.551	−0.640	0.343	3.460

SE, standard error; *β*, standardized partial regression coefficient; CI, confidence interval; VIF, variance inflation factor.

As shown in Figure 1B, participants with SCZ tended to accumulate more visceral fat than those with other psychiatric diagnoses, possibly due to pharmacological side effects (e.g., increased appetite) and dietary choices rich in simple carbohydrates and saturated fats, despite having access to balanced institutional meals.

Figure 1C shows no significant association between CRP, body fat, and diagnosis (*p* = 0.253). Moderate and marked CRP elevations were observed in 62.8 and 14.2% of participants, respectively, while only 23.0% had normal CRP values; there were no group differences (Table 4). In the S group, 20.4% were overweight and 6.2% were obese (Table 4).

In general, considering the median age (58 years) and BMI (24.14 kg/m^2^), the sample presented a normal body weight range with a risk of overweight. According to the linear regression analysis, there was no significant relationship between CRP and BMI (*p* = 0.305; Figure 1D).

Figure 1 presents scatter plots comparing biochemical and anthropometric variables between participants with SCZ and those without. Panel A shows the relationship between clinical diagnosis, creatinine, and age; panel B between clinical diagnosis, visceral fat, and age; panel C between CRP, body fat, and diagnosis; and panel D between CRP, BMI, and diagnosis.

## 4 Discussion

This study shows a high prevalence of anemia, low vitamin D levels, low HDL cholesterol, high total cholesterol, low bone mass, and low serum creatinine levels. Visceral fat and body fat percentage were variables that have a high impact on nutritional status and should be constantly monitored. According to the GLIM criteria, including phenotypic components (weight loss, low body mass index, and reduced muscle mass) and etiological components (reduced food intake or assimilation and inflammation), none of our participants met the definition of malnutrition.

These results are noteworthy because the external catering service provided nutritious meals to the patients, and most of them spent several hours physically active and exposed to sunlight. A study from China found that the rate of anemia in patients with CZS was 26.36% (18). A study in a Palestinian population found that 55.9% of women and 13.7% of men with CZS suffered from anemia (19). Furthermore, chronic psychiatric patients in Turkey had a high prevalence of anemia (25.4%), including BD, psychotic disorder, conversion disorder, obsessive-compulsive disorder, generalized anxiety disorder, and other psychiatric conditions. It was also determined that women (35%) with SCZ suffer more from anemia than men (10%) with the same disorder (20).

The frequency of anemia was highest among patients with a psychotic disorder (35%). Vitamin B12 deficiency may be related to psychiatric symptoms and imbalance. Previous studies have shown that vitamin B12 deficiency can lead to psychiatric symptoms (21–24).

The prevalence of anemia and vitamin D deficiency in patients with SCZ may vary substantially across regions due to differences in dietary intake, sun exposure, healthcare infrastructure, and sociocultural factors. In Qatar, recent cross-sectional studies reveal suboptimal levels of hemoglobin, ferritin, and vitamin D among patients with SCZ, with inflammatory and metabolic abnormalities, such as BMI (25–28). Elevated HbA1c and triglycerides are prominent, especially in treatment-resistant cases. These metabolic alterations, potentially driven by antipsychotic-induced weight gain and chronic low-grade inflammation, may impair iron metabolism and reduce vitamin D bioavailability. Furthermore, intense sun avoidance due to cultural dress codes and limited nutritional screening programs worsens micronutrient deficiencies. Together, these findings highlight the critical role of integrative and context-specific nutritional surveillance in the care of SCC.

Anemia-induced cerebral hypoxia and oxidative stress impair neurotransmitter metabolism, particularly serotonin and glutamate, increasing the risk of suicide. A recent longitudinal study of major depressive disorder identified a low baseline red blood cell count as a significant predictor of suicidal ideation trajectories, suggesting erythrocyte-mediated modulation of neuroinflammatory circuits (e.g., IL-6 and TNFα). These findings imply that anemia and decreased red blood cell function contribute to suicidality by disrupting oxygen delivery and inflammatory pathways. The overall prevalence of serum 25(OH) D < 75 nmol/L (21.63 ng/mL) was estimated to be 76.6% between 2000 and 2022 (29).

This result is similar to that of a study published in the *New England Journal*. Vitamin D deficiency is a major challenge worldwide and does not appear to be exclusive to patients with multiple sclerosis (30). A study conducted in the Netherlands, which evaluated serum vitamin D levels in 118 participants, showed that 65.8% of those diagnosed with SCZ had low vitamin D levels (≤12 ng/mL: deficient + insufficient) (31). Another study, conducted between 2018 and 2022 in Ecuadorian cities, such as Quito, Ambato, Ibarra, and Santo Domingo, reported that approximately 68.8% of participants from the general population had serum 25(OH) D levels < 30 ng/mL (32).

Several studies have reported a high prevalence of fracture risk and osteoporosis in patients with SCZ (14, 33). Marthoenis et al. found that compared with healthy individuals, patients with SCZ had a higher prevalence of underweight and a lower prevalence of overweight and obesity. They also reported that SCZ diagnosis was significantly associated with reduced muscle mass, reduced bone mass, higher basal metabolic rate, older metabolic age, and increased total body water (34). In contrast, Tang et al. found no evidence to support a definitive association between mental illness and osteoporosis risk, contradicting many previous observational studies (35).

Risk factors for hepatic steatosis are similar in both the general population and patients with SCZ (36). Patients with SCZ have increased intra-abdominal fat, which could explain their premature death (37). A study conducted in Russia revealed that the median body fat in 110 patients with CZS without metabolic syndrome was 29.9% (38). Marthoenis et al. showed a mean of 25.1 and 7.6% for the percentage of body fat and visceral fat in patients with multiple sclerosis (CZS), similar to our results (34).

Regarding low serum creatinine levels, one possible factor is the occurrence of pharmacological side effects resulting from the use of specific medications or interactions with others. It is possible that psychiatric illnesses may cause indirect damage to muscle tissue, the liver, or the kidneys through as yet unidentified mechanisms, resulting in abnormal serum creatinine levels.

Although the majority of patients have general diets (89% in our sample), INC dietitians indicate specialized diets depending on the case. Most patients use the entire area (about 200 square meters for men and the same for women) to walk at their individual discretion. They sporadically leave the area with technical support. They have a markedly sedentary life, which could contribute to the worsening of their condition.

Quetiapine and chlorpromazine, via H1/5HT2C receptor antagonism, potentiate SREBP1c–driven *de novo* lipogenesis, increasing visceral adiposity. Carbamazepine induces CYP2R1 and other P450s, accelerating 25-hydroxyvitamin D catabolism, thereby lowering its serum levels. Pregabalin and biperiden have minimal metabolic impact, whereas levomepromazine shares anticholinergic effects that can alter appetite. Levothyroxine elevates basal metabolic rate, preserving lean mass; metformin activates AMPK, mitigating insulin resistance and visceral fat; carvedilol modulates lipid oxidation and oxidative stress; losartan’s AT1R blockade reduces inflammation and may normalize creatinine. Chronic IL6 and TNFα excess further impair iron homeostasis and bone turnover.

Our study integrates anthropometric measures and bioimpedance derived body composition analysis with detailed biochemical profiling in adults with chronic SCZ. Its innovative design not only addresses nutritional disparities within the target region but also evaluates visceral adiposity, BMD, nutrient status, and renal biomarkers to inform comprehensive clinical care strategies.

The limitations of our study include a small sample size and a lack of tests related to creatinine. Further studies could explore creatinine using tests such as glomerular filtration rate, creatinine clearance, and albumin–creatinine ratio. The cut-off points for body composition are based on data from the US and Europe, rather than Latin America. Additional testing, including dual-energy X-ray absorptiometry, procollagen I N-terminal propeptide, and osteocalcin, among other potential candidates, could facilitate a more comprehensive understanding of bone health. Moreover, the secondary effects of drugs on the body, diets, sample sizes, and epigenetic mechanisms require further investigation.

Here we suggest some recommendations. In chronic SCZ care, mental health clinicians should:

1. Conduct routine nutritional and metabolic assessments, such as complete blood count with ferritin, serum 25-hydroxyvitamin D [25(OH)D], and lipid panels, and provide targeted supplementation as indicated.
2. Prescribe structured medical nutrition therapy alongside aerobic and high-intensity interval training to reduce visceral adiposity.
3. Engage endocrinology, dietetics, and physiotherapy specialists in an integrated multidisciplinary approach to optimize metabolic parameters and clinical outcomes.

## 5 Conclusion

The findings of this study highlight the high prevalence of anemia, low vitamin D levels, low HDL, high total cholesterol, low bone mass, and low serum creatinine in individuals with chronic SCZ and other mental illnesses. These results emphasize the need for targeted nutritional interventions to address these deficiencies and improve overall health outcomes. The significantly higher visceral fat accumulation in patients with SCZ suggests that metabolic alterations, possibly linked to antipsychotic medication use and dietary habits, could contribute to increased cardiovascular and metabolic risks.

Given the markedly sedentary lifestyle observed in this population, structured physical activity programs should be incorporated into treatment plans to mitigate the risks associated with obesity, metabolic syndrome, and osteoporosis. Future research should explore the molecular mechanisms underlying metabolic alterations in SCZ, the long-term impact of nutritional deficiencies on cognitive function, and the effects of psychiatric medication on metabolic health. A multidisciplinary approach, including psychiatric, nutritional, and physical health interventions, is essential to improve the quality of life for individuals with chronic SCZ.

## Data Availability

The original contributions presented in the study are included in the article/supplementary material, further inquiries can be directed to the corresponding author.
